# Chemical and Biological Characterization of Oleanane Triterpenoids from Soy 

**DOI:** 10.3390/molecules14082959

**Published:** 2009-08-10

**Authors:** Wei Zhang, David G. Popovich

**Affiliations:** Department of Chemistry, National University of Singapore, Science Drive 4, 117543, Singapore

**Keywords:** oleanane triterpenes, soy, *Glycine max*, Hep-G2, apoptosis, MTT

## Abstract

Soyasaponins are a group of complex and structural diverse oleanane triterpenoids found in soy (*Glycine max*) and other legumes. They are primarily classified into two main groups − group A and B − based on the attachment of sugar moieties at positions C-3 and C-22 of the ring structures. Group A soyasaponins are bidesmosidic, while group B soyasaponins are monodesmosidic. Group B soyasaponins are further classified into two subcategories known as 2,3-dihydro-2,5-dihydroxy-6 -methyl-4H-pyran-4-one (DDMP) and non-DDMP conjugated molecules. The preparation and purification of soyasaponin molecules is complicated by the presence of bioactive soy isoflavones, which often overlap with soyasaponin in polarity and must removed from extracts before biological assessment. Soyasaponin extracts, aglycones of group A and B and individual group B soyasaponins such as soyasaponin I have been reported to posses specific bioactive properties, such as *in vitro* anti-cancer properties by modulating the cell cycle and inducing apoptosis. The isolation, chemical characterization and detection strategies by HPLC and HPLC-MS are reviewed, along with the reported bioactive effects of soyasaponin extracts and individual molecules in cultured cancer cell experiments.

## Introduction

### Soyasaponin classification

**Figure 1:**
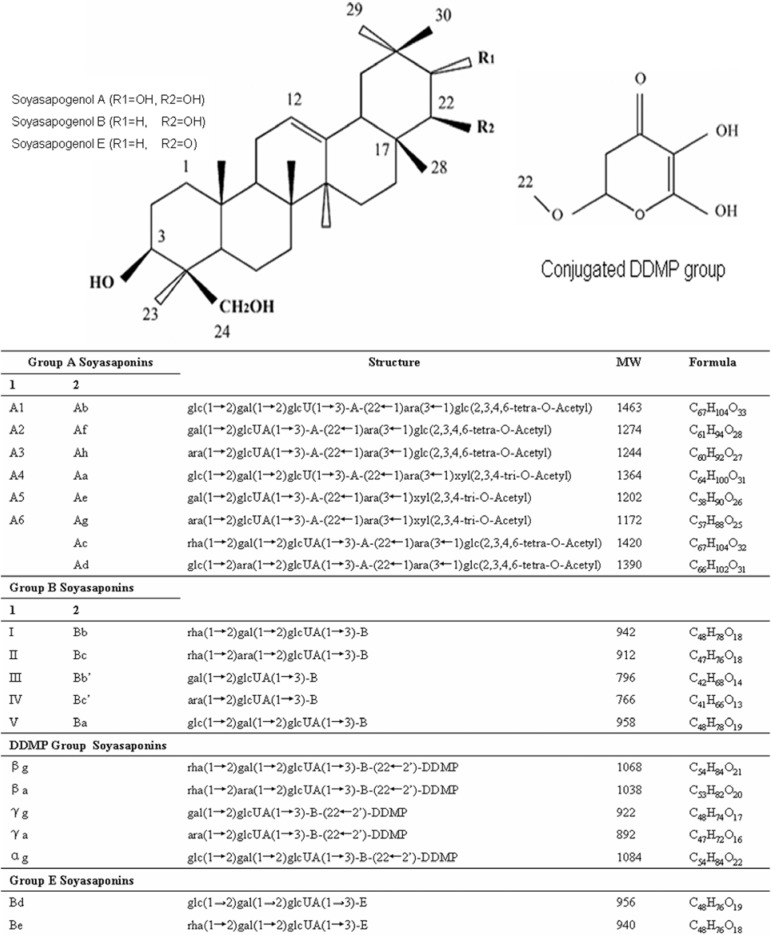
The chemical structures and molecular weights of soyasaponins.

Soyasaponins are oleanane triterpenoid glycosides possessing complex and diverse structures. They are found in soy (*Glycine max*) and other legumes, such as green peas (*Pisum sativum* L) and lentils (*Lens culinaris*) [1,2,3]. Soyasaponins are amphiphilic molecules, with polar water soluble sugar moieties attached to a nonpolar, water insoluble pentacyclic ring structure. Soyasaponins are categorized according to the individual aglycones (soyasapogenols), and there are two main aglycones, referred to as group A and group B, respectively. Group A soyasaponins are bidesmosidic saponins with two glycosylation sites at carbons 3 and 22 on the oleanane ring structure. Group A soyasaponins can be further divided into two groups, known as acetylated and deacetylated forms [4,5]. Group B soyasaponins have one glycosylation site on their aglycones (carbon 3) and are also classified into two groups, based on the conjugation at carbon 22 with a 2,3-dihydro-2,5-dihydroxy-6-methyl-4-pyrone (DDMP) moiety or non-DDMP conjugated soyasaponins. DDMP conjugated soyasaponins are known as αg, βa, βg, γa and γg, and non-DDMP conjugated soyasaponins are known as soyasaponins I, II, III, IV and V [5,6,7]; the chemical compositions are shown in Figure 1. DDMP group B soyasaponins are thought to be the more abundant group of soyasaponins in soy [5]. There are two different naming conventions currently utilized in the literature, which adds to the complexity of summarizing and interpreting the reported literature [3,4,5,8] and both conventions are shown in Figure 1. There is a third soyasaponin aglycone known as group E, which has a ketone at position C22 and has been reported to be formed during soy extraction [8,9,10,11]. Group E soyasaponins Bd and Be have been reported to be transformed into group B aglycone during acid hydrolysis [12], thus suggesting that the group E aglycone might be an artifact formed during alcoholic soyasaponin extraction [13] and not be naturally occurring. Generally, plants store saponins as glycosides, typically in the bidesmosidic form, that can be hydrolyzed to the monodesmosidic forms and this change from bidesmosidic to monodesmosidic has been reported to enhance the bioactivity of saponins [14]. 

Soyasaponins are thought to be bioactive molecules, and there are many reports relating the bioactive response to the soyasaponin structure [8,9,15,16]. In this review, the isolation, chemical characterization and detection strategies, focusing on HPLC and LC-MS, to analyze soyasaponins will be discussed, along with the reported bioactive effects of soyasaponins extracts and individual molecules assessed in cultured cancer cell experiments.

### Soyasaponin extraction

Although scientific reports on soyasaponins have been around for at least 80 years [17], the extraction, purification and quantification still presents many challenges. Soyasaponin glycosides are structurally similar and can possess overlapping HPLC retention times, and similar molecular masses and fragmentation patterns when analyzed using mass spectrometry (MS); furthermore, some soyasaponins such as the group B DDMP conjugated are heat labile [7,18,19], further confounding authentic saponin identification and quantification. 

DDMP refers to 2,3-dihydro-2,5-dihydroxy-6-methyl-4H-pyran-4-one (DDMP), glc, β-D-gluco-pyranosyl; gal, β-D-galactopyranosyl; glcUA, β-D-glucuronopyranosyl; ara, α-L-arabinopyranosyl; rha, α-L-rhamnopyranosyl; xyl, β-D-xylopyranosyl; MW, molecular weight. Columns numbered 1 and 2 refer to the different naming conventions [4,5].

The purification process is complex and laborious, with long sample preparation and extraction times and yielding relatively low amounts of soyasaponins from soy [3]. The amount of soyasaponins found in soy and related products are generally between 1.8% and 4.4%, depending on the variety and cultivation conditions of the soybeans [3]. The lack of a full complement of available commercial soyasaponin standards [20] has slowed the pace of research. Many of the current studies on soyasaponins have focused on extraction and analysis, usually producing relatively low yields and in insufficient quantities for biological activity testing of individual compounds.

Conventional extractions and preparation of soyasaponins from defatted soy flour or soy products typically involves the use of organic solvents, usually either aqueous ethanol or methanol with agitation at room temperature. Room temperature extraction is needed to ensure extraction and to prevent the breakdown of the DDMP conjugated compounds and most of the sugar glycoside molecules [21]. The extraction efficiency is dependent on three main factors: time, temperature (both ambient and solvent temperature) and choice of solvents [18,19,20,22]. The optimum extraction time has been reported to be between 4 to 6 h for the maximum yield of soyasaponins from soy flour using absolute methanol at 60 ^o^C under reflux [23]. We have recently compared four different aqueous methanol extractions techniques: room temperature for 24 and 48 h with constant agitation, refluxing in methanol for 4 h at 60 °C, ultrasonic extraction, and Soxhlet extraction [19]. All four extraction techniques were suitable for extracting soyasaponins, but to different degrees. The Soxhlet extraction in methanol yielded significantly lower amounts of soyasaponins, compared to all other extraction techniques, while room temperature stirring in methanol yielded significantly higher amounts and an extraction time of 24 h was sufficient. The amounts of soyasaponins extracted from lowest to highest were found to be Soxhlet < reflux < sonication < 24 h stirring room temperature = 48 h [19]. A possible explanation for the observed differences in extraction may lie in the addition of thermal energy during the extractions, which can cause deglycosylation of saponin glycosides compared to room temperature extraction. Thermal energy likely transformed the DDMP conjugated group B soyasaponins such as bg into the corresponding non-DDMP soyasaponin I [7]. A similar funding was also reported for the extraction of dammarane triterpenoids saponins from ginseng (*Panax quinquefolius*) in boiling water [24]. 

### Soyasaponin extract preparation

The basic extraction methodology used to extract soyasaponins typically involves preparing crude extracts, then employing a column chromatography concentration followed by purification and separation of individual soyasaponins [18,20,22]. However, soyasaponins are not the only groups of biologically active molecules found in soy and related products. Soy’s biological activity can be attributed to three main classes of compounds: soy protein, isoflavones and the soyasaponins. Soy protein can be easily and selectively removed by ammonium acetate precipitation, however the isoflavones and the soyasaponins share overlapping polarities, making separation, quantification, and assessment of bioactivity difficult [19]. Employing column chromatography has been reported to be successful in separately the isoflavones from the soyasaponins. Decroos *et al.* [20] utilized an ethanol conditioned XAD-2 column to concentrate soyasaponins and isoflavones in defatted soy hypocotyls followed by fractioning and purification using preparative scale reverse phase chromatography [20]. The preparative chromatographic approach was shown to be effective in separating the isoflavones from the soyasaponins, based on different retention times, as the isoflavones were reported to elute earlier than the soyasaponins [20]. However, the purities of individual soyasaponins Ab, αg and βg were not optimal and required a second chromatographic step [20]. Preparative HPLC is an effective procedure to separate individual soyasaponins, but it is hampered by the large amounts of solvent needed and the low recovery yield. Semi-preparative purifications have also been successfully utilized for soyasaponin purification [22]. Alternatively, utilization of solid phase extraction has been reported to be a simple and economical alternative to achieve relatively pure soyasaponins between 85-90% [23]. We have recently reported a robust method based on solid phase extraction (SPE) utilizing various concentrations of methanol to separate the isoflavones from the soyasaponins, separate group B soyasaponins from group A and to produce relatively pure extracts [19,25]. A concentration of 50% methanol was found to be the optimum to separate the group B soyasaponins from the isoflavones, using SPE, virtually removing all but less than 1% of the isoflavones [19].

### Analysis and determination of soyasaponins

There are various reported methods for the determination of soyasaponins from soy and soy products. Separation by thin layer chromatography and quantification using a densitometer has been reported as an economical and effective way to separate and quantify soyasaponins [26], but high-performance liquid chromatography (HPLC) utilizing a reversed-phase column is the most prevalent analytical technique for soyasaponins analysis. Various detectors have been used, such as ultraviolet or photo diode array (PDA) [6,18,27], and evaporative light scattering detection (ELSD) [12,28]. The maximum absorption wavelength of most of soyasaponins is a about 205 nm [22], while some of the DDMP soyasaponins can reach 295 nm. Due to the large number of soyasaponin glycosides found in soy, the development of an all encompassing UV detection method is difficult to achieve [27] and often two different gradient programs are utilized to achieve full soyasaponin detection. An analytical method using UV was reported for the detection of all the known group B soyasaponins at a wavelength of 205 nm, provided standards have been prepared in advance [18]. ELSD, which is based on mass detection by light scattering after evaporation of the mobile phase, has been successfully used for the detection of soyasapogenols, soyasaponins and ginseng saponins [29]. Rupasinghe *et al.* [12] reported ELSD could detect the authentic soyasapogenols. However, ELSD like UV, has some disadvantages such as an extensive sample preparation and potential interference when detecting low quantities in serum [30]. Often one solvent gradient program is optimized to separate the more abundant soyasaponin glycosides and one is optimized for the separation of the soyasapogenols leading to a time consuming analysis [16,19]. Table 1 and Table 2 lists the various HPLC analysis strategies employed in the recent literature to detect the soyasaponin glycosides and the soyasapogenols respectively.

**Table 1:** Concentration, HPLC Quantification Methods of Soyasaponins.

Concentration Method [ref.]	Group A	Group B	Column	Group A Solvent Program	Group B Solvent Program
XAD-2 [29]	Ab, Non-acetylated Ab,	Ba, Bb, αg, βg	Source 15 RP	Gradient A: Water with 0.001% acetic acid (v/v); B: Acetonitrile with 0.001% acetic acid (v/v)	Gradient A: Water with 0.001% acetic acid (v/v); B: Acetonitrile with 0.001% acetic acid (v/v)
Silica gel [22]	All	All	Semi-Preparative Waters íBondapak C18 column	Isocratic Methanol, 2-propanol, water and formic acid (45:5:50:0.1) (v/v)	Isocratic Methanol, 2-propanol, water, and formic acid (55:5:40:0.1) (v/v)
Flash chromatography system [31]	All	All	Preparative HPLC system Luna C18(2) column	Pre-equilibrated Acetic acid, acetonitrile, and water (1:30:69) (v/v). Gradient A: 100% Acetonitrile B: Water	Pre-equilibrated Acetic acid, acetonitrile, and water (1:30:69) (v/v). Gradient A:100% Acetonitrile B: Water
SPE [15,19]	All	All	SPE	Water and Methanol	Water and Methanol
C18 Lobar column [18]	NA	All	Semi-preparative HPLC systemRP-18 column		Isocratic DDMP: Acetonitrile, water, TFA (40:59.95:0.05) (v/v) NON-DDMP: Acetonitrile, water, TFA (36:63.95:0.05) (v/v)
C18 Cartridge [32]	NA	Ba and Bb in human serum	HPLC-MS system In MRM mode XDB-C18 column		Gradient A: 0.025% AcOH in water (v/v); B: 0.025% AcOH in MeCN (v/v)

**Table 2:** Quantification Methods for Soyasapogenol A and B.

Compound	MW	Formula	Analysis Method	Solvent System and Program	Specification
Soyasapogenol A	474	C_30_H_50_O_4_	TLC [33] Silica gel 60G	Light petroleum (b.p. 60-80 °C), ethyl acetate (4:3) (v/v)	Visualization 10% sulfuric acid in ethanol and viewing under UV
			TLC [34]	Dichloromethane and methanol (9:1) (v/v)	Spraying with a saturated solution of potassium dichromate in sulfuric acid
			Normal HPLC [33]	A: Light petroleum (b.p. 60-80°C); B: Ethanol, 0-7.5min, 0-7.5% B; 7.5-15 min, 7.5% B isocratic; 15-20 min, 7.5-20% B	Silica Column (250mm × 4.6mm) Flow-rate 1.5 mL/min
Soyasapogenol B	458	C_30_H_50_O_3_	Revised HPLC [12]	Acetonitrile: 1-propanol: water: 0.1% acetic acid (80:6:13.9:0.1) (v/v) Isocratic	ODS C18 column (250mm × 4.6mm) Flow-rate 0.9mL/minELSD detection
			Revised HPLC [35]	A: Acetonitrile: 1-propanol: water: acetic acid (80:6:13:0.1) (v/v); B: 100% Acetonitrile 0-15 min 100% A isocratic; 15-17 min 0-100% B; 17-19 min 100% B; 19-22 min back to 100% A	RP-C18-AB column (250 × 4.6 mm) Flow-rate 0.9 mL/min

Identification of the soyasaponins of interest typically requires MS analysis, either on a HPLC-MS system or an individual MS to confirm the molecular weights. HPLC-MS detection seems to be most relevant and effective method for the identification of soyasaponins [32], other triterpenoids [36] and specifically group B soyasaponins [32,34]. Decroos *et al.* [29] developed an HPLC-ELSD-ESI-MS method for analysis all groups of soyasaponins, including acetyl soyasaponins group A and DDMP group B soyasaponins. MS detection of oleanane triterpenoids is complex, and requires experienced personnel and expensive equipment, which is usually not available for daily routine analysis in all laboratories [37]. Complicated fragmentation patterns are produced during ionization, resulting in molecular weight confirmation issues. Heftmann *et al.* [38] reported oleanane triterpenoid ring structures of the soyasapogenols are themselves prone to fragmentation due to a reverse Diels-Alder reaction [38]. For all these reasons MS analysis of the soyasapogenols can be challenging. For example, soyasapogenol A have been reported to produced a fragmentation pattern with the most abundant ion in positive mode corresponding to molecular weight of 250, while the corresponding soyasapogenol B ion was observed at 234 [38]. These two fragment are caused by the reverse Diels-Alder reaction and, correspond to molecular weights of 474 and 458 of the respective soyasapogenols [38]. In our laboratory we have optimized the HPLC-ESI procedure on a Thermo Finnigan LCQ-ESI quadrapole ion trap LC-MS (Thermo Fisher Scientific, USA) system and have been successful in determining the molecular ions for a number of group B soyasaponins such as I, III, βa, βg; the fragmentation patterns are shown in Table 4 [19]. Soyasapogenol fragmentation and breakdown during ESI analysis of group B soyasaponins was avoided by carefully adjusting the fragmentation temperature and generally keeping the internal temperature below 250 °C. Table 3 summarizes the recent literature on the detection and use of HPLC-MS technique for soyasaponin analysis. 

**Table 3:** LC-MS Analysis of Soyasaponins.

LC-MS System	LC-MS LC Program	MS Condition	LC-MS Mode	Detected Soyasaponins
Agilent 1100 series LC/MSD Trap SL [39]	Waters AccQ.Tag column A: 0.025% AcOH in water (v/v) B:0.025% AcOH in MeCN (v/v) temperature: 35°C flow-rate: 0.5 mL/min	ESI Negative mode Capillary voltage: 4.4 Kv Dry Temperature: 350 °C	MRM Transition Setting m/z: 958→940, 942→924 and 822→351	Ba, Bb in serum
Waters/Micromass Ultima LC/MS instrument, consisting of Waters 2690 liquid chromatograph with a Waters 996 PDA [40]	Zorbax Eclipse XDB-C18 column A: Water with 0.05% TFA (v/v) B: ACN with 0.05% TFA (v/v) flow-rate: 0.5 mL/min	ESI Positive mode Capillary voltage: 3.5 Kv Dry Temperature: 350 °C	Full Scan SIR quantification	Group A: Ab, Ac, Af Deacetyl Ab, Ac, Af Di-deacetyl Ab, Tetra-deacetyl Ab, Af Tri-deacetyl Ad Group B: Ba, Bb, Bb′, Bc, Bc DDMP Bb, Bc, Ba
Agilent LC/MSD Trap SL [32]	Waters AccQ.Tag column A: 0.025% acetic acid in water (v/v) B: 0.025% acetic acid in MeCN (v/v) temperature: 35 °C flow-rate: 1 mL/min	ESI Negative mode Capillary voltage: 4.4 Kv Dry Temperature: 350 °C	Full Scan SIR quantification	Group A: Aa-Af Group B: Bb, Bb’, Bd and Be DDMP αg, βg, βa, γg
Agilent 1100 series LC/MSD Trap SL [30]	The same as [39]	ESI Negative mode Capillary voltage: 4.4 Kv Dry Temperature: 350°C	Full Scan SIR quantification	Group A: Aa, Ab Group B: Ba, Bb, Bb’DDMP βg
Waters HPLC with Finnigan LCQ quadrupole ion trap MS with MSn [19]	Shimadzu reversed phase C-18 A: 2.5% acetic acid in water (v/v) B: 100% Acetonitrile Column temperature: 25 °C flow-rate:1 mL/min	ESI Positive and Negative Capillary voltage: 4.4 Kv Dry temperature: 200 °C	Full Scan	Group B: I, III, DDMP βg, βa, γg, γa Group E: Be
Bruker Esquire LC with ESI-MS system [41]	J.T.Baker C18 reverse column Linear solvent: 0.1% acetic acid in water/Acetonitrile 95:5 to 5:95 (v/v) in 90 min temperature: not reported flow-rate: 0.8 mL/min	ESI Negative Capillary voltage: 3 Kv Dry temperature: 360 °C	Full Scan SIR quantification	Group B: Soyasaponin I Soyasapogenol E and B
Waters 2690 Alliance HPLC system coupled with a Micromass Mass spectrometer [22]	Supelcosil LC-18-DB column A: 0.2% formic acid in water (v/v) B: 0.2% formic acid in Methanol (v/v) temperature: not reported flow-rate: 1mL/min	ESI Negative Capillary voltage: 3.7 Kv Dry temperature: 200 °C	Full Scan SIR quantification	Group A: Aa, Ab, Ac, Ae, Af, Ag and Ah Group B: Ba, Bb, Bc, Bb’, Bc’, Bd Group E: Be
Dynamax Model SD-200 with Hewlett-Packard HP5898 B quadrupole Mass Spectrometer [42]	SupLC-18 microbore column A: 30% Acetonitrile in water (v/v) B: 100% Acetonitrile temperature: not reported flow-rate: 0.1 mL/min	ESI Positive and Negative Capillary voltage: not reported Dry temperature: 150 °C	Full Scan	Group B: I, II and V DDMP βg Group A: Acetylsoysaponin A4
**FAB MS System**	**FAB MS Condition**		**FAB MS Mode**	**Detected Soyasaponins**
JEOL JMS SX 102/102 high-resolution double-focusing four-sector tandem mass spectrometer (FAB/MS) [42]	Full accelerating voltage of 10 keV Resolving power 3 x 10^3^ Xenon was used for providing fast atoms 20 mA discharge current Magnet scan rate: 10s per decade		Positive and Negative Full Scan MS/MS Detection	Group B: I, II and V DDMP βg Group A: Acetyl soyasaponin A4

### Hydrolysis

Much of the early work on soyasaponins and other triterpenes such as the dammarane saponins from ginseng utilized acid or alkaline hydrolysis to deglycosolate the sugar moieties of the triterpene, leaving for the most part an intact aglycone [43]. The aglycone and related glycosides were then separated and visualized on thin-layer chromatography (TLC) before and after hydrolysis to confirm the presence of different aglycones and to assign new compounds to their respective groups based on TLC migratory patterns. 

Acid hydrolysis is generally the preferred method for preparing soyasapogenols from crude soyasaponin extracts [13,16,26,37]. Acid hydrolysis in anhydrous methanol has been reported to enable the highest recovery of soyasapogenols A and B without producing artifacts [12,13]. Moreover, Ireland and Dziedzic [13] showed that hydrolysis for 3 h with 3% sulfuric acid in anhydrous methanol produced the greatest yield of soyasapogenols and anhydrous methanol has also been shown to increase the yield during acid hydrolysis [12]. 

Alkaline hydrolysis on the other hand, tends to produce partial hydrolysis of soyasaponins. Partial alkaline hydrolysis has been reported useful for preparing non-acetylated group A soyasaponins and non-DDMP group B soyasaponins [22,23]. Acetyl group A soyasaponins were reported to converted to non-acetyl soyasaponins A1 and A2 during saponification using alkaline treatment. DDMP conjugated group B soyasaponins are easily cleaved in alkaline conditions [6,19], [16] resulting in their corresponding non-DDMP molecule. Gurfinkel *et al.* [26] found the relative proportion of saponified soyasaponins significantly increased after alkaline treatment except for soyasaponin III. When compared to acid hydrolysis, alkaline treatment can assist with the cleavage of the DDMP conjugation without affecting the glycoside bond at position C-3 of the ring structure [19]. The DDMP moiety of group B soyasaponins has been found to be cleaved from soyasaponins easier than compared to the acetyl groups of group A soyasaponins [22]. In our laboratory we have utilized a characteristic partial alkaline hydrolysis (5% NaOH) in anhydrous methanol to produce an extract of approximately 65% soyasaponin I and 29% soyasaponin III [19] in sufficient amounts for cellular studies.

**Table 4:** Selectivegroup B soyasaponins ion fragments separated and analyzed by LC-MS [19].

Soyasaponin	Mass	Ion Fragments, m/z	
		[M+H]_­­_^+^	Others
I	942	943.1	1045.9, 945.2, 944.1, 913.1, 531.7, 142.7
II	912	913.1	1045.0, 1014.2, 944.1, 914.1, 532.0, 516.7, 142.6
III	796	797.2	1015.0, 914.0, 913.1, 799.2, 142.6
IV	766	767.1	913.1, 769.1, 536.2, 464.4, 142.6
V	958	959.2	1029.2, 961.2, 960.2, 519.2, 142.7
βg	1068	1069.2	911.0, 594.6, 142.8
βa	1038	1039.0	795.2, 579.8, 142.6
γg	922	923.2	924.2, 925.2, 501.6, 142.6
γa	892	893.2	894.1, 923.2, 924.1, 1012.8, 566.6, 527.2, 142.5
**Be**	940	941.1	942.1, 531, 142.8

### Measured bioactivities of soyasaponins in cell culture

Much of the reported bioactivity attributed to soyasaponins has utilized crude alcoholic extracts derived from defatted soy. Crude or total soyasaponin extracts are typically mixtures of group A and B soyasaponins with small amounts of soyasapogenols [16]. The biological activity of each individual saponin is currently unknown, although progress on the separation and isolation of sufficient quantities and bioactive testing is being made in our laboratory [19] and elsewhere [44,45]. Production of sufficient quantities of active extracts utilizing a bioassay guided fractionated approach extracts is a complex process [25] and the bioactivity of soyasaponins largely depends on their respective chemical structures. The presence of water soluble sugar moieties, DDMP and acetyl groups in the soyasaponin structures affects the polarity, which may mitigate changes in bioactivity [46]. Many studies have shown that mixtures of soyasaponins have measurable bioactivity in cell culture studies. Gurfinkel and Rao [9] reported that there was a relationship between structure and bioactivities with soyasapogenols A and B generally being more bioactive compared to the glycosides [9]. Research on the structure- activity relationships involving soyasaponins is ongoing. There is some evidence, as with many other saponins, that bioactivity of soyasaponins increases as sugars moieties are eliminated from the saponin structure, thereby reducing the polarity [14]. The position of the glycosides is also likely to determine certain bioactive functions such as membrane permeability. This has been demonstrated using dammarane saponins of ginseng [47]. Two molecules with similar chemical makeup, except for the location of a glucose moiety either at carbon 3 (Rh2) or carbon 6 (Rh1) had vastly different effects in cultured leukemia cells. Ginsenoside Rh2 was found induce apoptosis and concurrently increasing membrane permeation compared to Rh1 [47]. We are currently pursing bioactive classification of soyasaponins using cultured hepatocarcinoma cells and have begun with group B soyasaponins with the aim of providing evidence of structure function relationship for these molecules.

Recent studies have shown that a total soyasaponin extract can inhibit the growth of Hela (cervical tumor) cells [44], Hep-G2 (hepatocarcinoma) cells [16], and in colon adenocarcinoma cells (HCT-15) [45,48] by inducing programmed cell death, either apoptosis or microautophagy. Apoptotic processes remove damaged or mutated cells and recycle the cellular components [49]. Total soyasaponin extract prepared from defatted soy flour was reported to reduced the growth of cultured Hep-G2 cells after 72 hours of treatment measure by the MTT (3-(4,5-dimethylthiazol-2-yl)-2,5-diphenyltetrazolium bromide) assay [16]. The LC_50_ value was determined to be 0.6 mg/mL. In Hela cells after four days of treatment with a total soyasaponin extract the LC_50_ was estimated to be 0.4 mg/mL [44]. In both studies a total soyasaponin extract reduced cell growth through the induction of apoptosis. In Hela cells after four days of treatment, total soyasaponins (0.4 mg/mL) showed an increase in sub-G1 cells (apoptotic cellular fragments) of 10% during cell cycle analysis and 9% in Hep-G2 cells [16,44]. Xiao *et al.* [44] found that soyasaponin treatment reduced mitochondrial transmembrane potential and increased the intracellular Ca^2+^ concentration leading to apoptosis [44]. Total soyasaponin extracts have also been reported to reduce colon carcinoma cell growth in a number of studies [50,51,52,53]. Soyasaponins were reported to modify the cell membrane of cultured cells [54], potentially increasing membrane permeability. Increased membrane permeability has also been reported for the dammarane triterpenes derived from ginseng in both cultured leukemia and intestinal cells [47,55]. 

**Figure 2:**
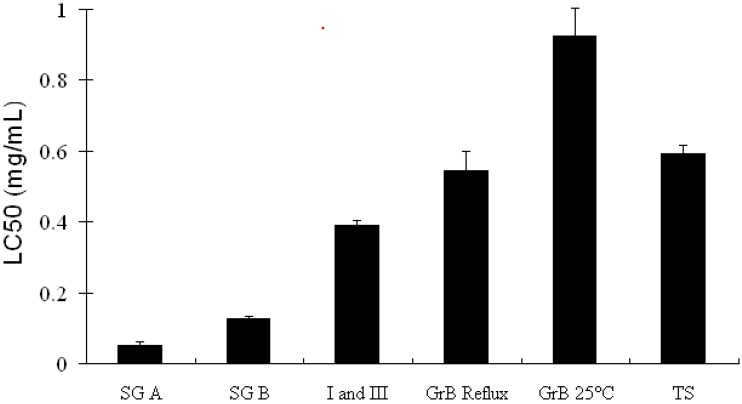
The effect of various soyasaponin extracts on hepatacarcinoma cells line (Hep-G2) viability after 72 hours of treatment.

Soyasapogenols prepared by acid hydrolysis were reported to inhibit the growth of Hep-G2 cells in a dose-dependent manner. The LC_50_ concentrations were determined to be 0.05 ± 0.01 mg/mL for soyasapogenol A and 0.13 ± 0.01 mg/mL for soyasapogenol B (Figure 2) [16]. Soyasapogenol A inhibited the growth of estrogen-insensitive human breast cancer cells (MDA-MB-231) at a concentration of 10 μM but stimulated the proliferation of estrogen sensitive cells (MCF-7) 2.5 fold [56]. Additionally, the ER-ERE DNA complex, a marker of estrogen activation, was induced by soyasapogenol A. Soyasapogenol B reduced the growth of MDA cells without a significant effect on MCF-7 cells at all concentrations tested [56]. 

The composition of the extracts were as follows: SG A contained soyasapogenol A (69.3%), SG B contained soyasapogenol B (46.2%), I and III contained 65% soyasaponin I and 29% soyasaponin III, Gr B reflux refers to a group B reflux prepared extract that contained I (32.9%), III (14.2%), Be (4.2%), βg (28.3%), βa (9.7%), γg (1.7%), <1% γa and 3% soyasapogenol B, Gr B 25°C refers to a room temperature methanolic extract consisting of I (10.4%), III (4.6%), Be (6.2%), βg (46.6%), βa (16.9%), γg (4%), γa (1.7%) and < 1% soyasapogenol B. The TS extract refers to a total soyasaponin extract with an unknown saponin composition [16,19,25].

### Soyasaponins and apoptosis

**Figure 3:**
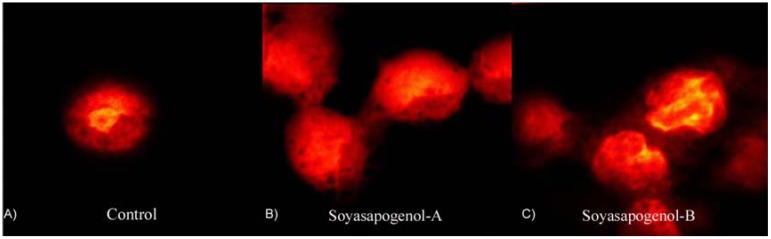
Confocal laser scanning images of propidium iodide stained hepatocarcinoma (Hep-G2).

Both soyasapogenol A and B containing extracts have been reported to be able to induce apoptosis. Soyasapogenol A extract treated Hep-G2 cells induced 47 ± 3.5% of the cells to undergo apoptosis while soyasapogenol B extracted induced 15 ± 4.2% after 72 h treatment. Apoptotic fragments were confirmed by confocal laser scanning images showing evidence nuclear condensing (pyknosis) and fragmentation (karyorrhexis) consistent with the apoptotic program cell death [16] and representative sample images is shown in Figure 3. Yanamandra *et al*. [57] demonstrated that group B soyasaponins had pro-apoptotic and anti-invasive activities in human glioblastoma cells (SNB 19). A well characterized group B extract, containing mainly soyasaponins I, II, III, and IV reduced cell invasion 45% compared to untreated cells measured by an *in vitro* invasion assay. Furthermore, a loss of mitochondrial trans-membrane potential was measured along with increase release of cytochrome C and increased caspase activity [57]. Five different soyasaponin extracts were tested and the accumulation of sub-G1 apoptotic cells measured by flow cytometry [16,25]. Generally, soyasapogenol A containing extracted showed the greatest propensity to affect the cell cycle compared to soyasapogenol B containing extract tested at the LC_50_ concentration compared to a fractionated extract or a total saponin mixture [16,25]. 

Cells were treated with soyasapogenol A (0.05 ± 0.01 mg/mL) panel (B) and soyasapogenol B (0.13 ± 0.01 mg/mL) panel (C) treated for 24 hours. Panel (A) represents untreated control cells [16].

Extract preparation can influence the bioactive response of soyasaponins. Two different group B extracts were prepared, one prepared by ethanol reflux of soy flour, which is rich in non-DDMP group B soyasaponins, and one prepared by room temperature extraction containing an abundance of DDMP conjugated soyasaponins. The major different between the two extracts was the DDMP conjugated soyasaponin βg [15,25]. The non DDMP soyasaponins reflux extract inhibited the proliferation of Hep-G2 cells to a greater extent than the room temperature DDMP soyasaponin extraction. The LC_50_ of the room temperature extract was found to be 0.9 ± 0.1 mg/mL and 0.5 ± 0.1 mg/mL for the reflux extract [15,25]. The reflux extract was found to induce apoptosis, as measured by the TUNEL assay, and affected the cell cycle progression whereas the room temperature extract induced differentiation of Hep-G2 treated measured by flow cytometry forward side scatter [15,25]. 

Soyasaponins have also been reported to induce macroautophagy, which is reported to be a type of programmed cell death [58]. Human colon cancer cells treated with soyasaponins suppressed proliferation, induced differentiation and inhibited protein kinase C activity [54]. Ellington *et al.* [45] reported that treatment of colon cancer cells (HCT-15) with an extract containing five different group B soyasaponins reduced cell growth after 24 and 48 h of treatment. Furthermore, treatment increased the percentage of cells in the S phase of the cell cycle while reducing cyclin-dependant kinase-2 (CDK-2) activity and a marker of macroautophagy (light chain 3) increased compared to non-treated cells. The induction of macroautophagy by group B soyasaponins was reported to be modulated by two important signaling pathway, group B treated cells were found to reduce Akt activity 50% affecting the phosphorylation of the ser^473^ phosphorylation increasing activity of ERK1/2 (MAPK) by 60% [48].

### Sialytransferase activity

Sialytransferase activity is associated with tumor metastasis and invasion [59,60]. Inhibition of sialytransferase activity is a useful target to delay the transformation of cells or slow the spread of metastasis. Wu *et al.* [61] showed that soyasaponin I was a highly specific inhibitor of *in vitro* sialytransferase activity [61]. Hsu *et al.* [62] confirmed that soyasaponin I was an *in vitro* sialytransferease inhibitor and was found to decrease α2,3-sialylations and ST3Gal IV expression which are important factors of the invasive behavior of tumor cells. Specifically, α2,3-linked sialic acids were suggested to play a role in the potential metastasis of murine melanoma cancer cell line B16F10 [63]. Soyasaponin I was found to specifically inhibit expression of α 2,3-linked sialic acids on the cell surface and to decrease the migration and cell adhesion to extracellular matrix proteins [63]. 

## Conclusions

Soyasaponins are a group of structurally complex bioactive molecules. The extraction, isolation, and purification processes are challenging and many attempts have been made to characterize the chemical and biological activity of soyasaponins from soy. Generally, soyasaponins can be extracted in native form by room temperature methanolic extraction and can be adequate separated and identified by HPLC-MS provided sufficient chemical standards or molecular weight data are available. In terms of biological activity, classification of these molecules suggests that soyasapogenols have greater *in vitro* cellular anticancer activity such as inducing apoptosis compared to the corresponding glycosides. However, soyasaponin glycosides such as soyasaponin I and III do posses biological activity and may mitigate changes in cancer cell properties by inducing cellular differentiation or inhibiting enzymes involved in metastasis. 
